# Carbolic Acid in Burns

**Published:** 1868

**Authors:** 


### CARBOLIC ACID IN BURNS.

Professor William Pirrie, of the University of Aberdeen, recommends carbolic acid and olive oil in the proportion of one to six as an application to burns. He relates in the *Lancet* the case of a delicate girl eleven years of age whose face, neck, side, back and arm were severely scalded by boiling water. Two folds of surgeon's lint were soaked in the carbolic acid and oil and applied over the whole surface, tin foil being placed above the lint to exclude the air. In ten minutes the patient was free from pain. On the second day the skin was greatly improved, and the bullæ which had formed seemed withering away. The skin was perfectly healed on the twelfth day, the cuticle having been thrown off. Not a drop of pus formed during the treatment.—*Pacific Medical Journal.*

